# Supplementary material for: H172Y mutation perturbs the S1 pocket and nirmatrelvir binding of SARS-CoV-2 main protease through a nonnative hydrogen bond
Source: Res Sq. 2022 Aug 9:rs.3.rs-1915291. Preprint. [Version 1] doi: 10.21203/rs.3.rs-1915291/v1 (PMC9387537; doi:10.21203/rs.3.rs-1915291/v1)
Supplement: Supplement 1 [file NIHPPRS1915291v1-supplement-1.pdf]

Supplementary Files

This is a list of supplementary files associated with this preprint. Click to download.

- MproH172YSI.pdf
